# Social, lifestyle, and health status characteristics as a proxy for occupational burnout identification: A network approach analysis

**DOI:** 10.3389/fpsyt.2023.1119421

**Published:** 2023-04-14

**Authors:** Fengshi Jing, Mengyuan Cheng, Jing Li, Chaocheng He, Hao Ren, Jiandong Zhou, Hanchu Zhou, Zhongzhi Xu, Weiming Chen, Weibin Cheng

**Affiliations:** ^1^Institute for Healthcare Artificial Intelligence Application, Guangdong Second Provincial General Hospital, Guangzhou, China; ^2^UNC Project-China, UNC Global, School of Medicine, The University of North Carolina, Chapel Hill, NC, United States; ^3^Guangzhou Baiyun International Airport Co., Ltd, Guangzhou, China; ^4^School of Information Management, Wuhan University, Wuhan, China; ^5^Nuffield Department of Medicine, University of Oxford, Oxford, United Kingdom; ^6^School of Traffic and Transportation Engineering, Central South University, Changsha, China; ^7^School of Data Science, City University of Hong Kong, Kowloon, Hong Kong SAR, China; ^8^School of Public Health, Sun Yat-Sen University, Guangzhou, China; ^9^Health Medicine Department, Guangdong Second Provincial General Hospital, Guangzhou, China

**Keywords:** occupational burnout, network science, health management, exponential random graph model, social networks

## Abstract

**Background:**

Occupational burnout is a type of psychological syndrome. It can lead to serious mental and physical disorders if not treated in time. However, individuals tend to conceal their genuine feelings of occupational burnout because such disclosures may elicit bias from superiors. This study aims to explore a novel method for estimating occupational burnout by elucidating its links with social, lifestyle, and health status factors.

**Methods:**

In this study 5,794 participants were included. Associations between occupational burnout and a set of features from a survey was analyzed using Chi-squared test and Wilcoxon rank sum test. Variables that are significantly related to occupational burnout were grouped into four categories: demographic, work-related, health status, and lifestyle. Then, from a network science perspective, we inferred the colleague’s social network of all participants based on these variables. In this inferred social network, an exponential random graph model (ERGM) was used to analyze how occupational burnout may affect the edge in the network.

**Results:**

For demographic variables, age (*p* < 0.01) and educational background (*p* < 0.01) were significantly associated with occupational burnout. For work-related variables, type of position (*p* < 0.01) was a significant factor as well. For health and chronic diseases variables, self-rated health status, hospitalization history in the last 3 years, arthritis, cardiovascular diseases, high blood lipid, breast diseases, and other chronic diseases were all associated with occupational burnout significantly (*p* < 0.01). Breakfast frequency, dairy consumption, salt-limiting tool usage, oil-limiting tool usage, vegetable consumption, pedometer (step counter) usage, consuming various types of food (in the previous year), fresh fruit and vegetable consumption (in the previous year), physical exercise participation (in the previous year), limit salt consumption, limit oil consumption, and maintain weight were also significant factors (*p* < 0.01). Based on the inferred social network among all airport workers, ERGM showed that if two employees were both in the same occupational burnout status, they were more likely to share an edge (*p* < 0.0001).

**Limitation:**

The major limitation of this work is that the social network for occupational burnout ERGM analysis was inferred based on associated factors, such as demographics, work-related conditions, health and chronic diseases, and behaviors. Though these factors have been proven to be associated with occupational burnout, the results inferred by this social network cannot be warranted for accuracy.

**Conclusion:**

This work demonstrated the feasibility of identifying people at risk of occupational burnout through an inferred colleague’s social network. Encouraging staff with lower occupational burnout status to communicate with others may reduce the risk of burnout for other staff in the network.

## 1. Introduction

Occupational burnout is a complex and multifaceted phenomenon that has become an increasingly prevalent concern in the field of psychology and psychiatry. It is defined as a psychological syndrome that results from prolonged exposure to chronic work stressors (1). From a psychological perspective, occupational burnout is often seen as the result of a mismatch between an individual’s personal characteristics and the demands of their work environment. From a psychiatric perspective, occupational burnout is often viewed as a form of depression or anxiety (2, 3). Such evidence suggests that although burnout is an occupational experience rather than a clinical diagnosis of mental health disorders, it does play a role in the onset of anxiety and depression symptoms, which are considered psychological ailments, among employees in the workplace. Individuals experiencing occupational burnout often report a sense of emotional exhaustion, depersonalization, and reduced personal accomplishment. These symptoms can lead to a range of negative outcomes, including decreased job performance, increased absenteeism, and reduced overall job satisfaction.

The consequences of occupational burnout are far-reaching and can have significant psychopathological, social, and economic implications. Psychopathologically, burnout can lead to an increased risk of mental health disorders such as depression and anxiety. Socially, burnout can lead to a breakdown in interpersonal relationships and increased social isolation. Economically, burnout can lead to decreased productivity, increased absenteeism, and increased healthcare costs. The cultural context in which occupational burnout is studied is also important to consider. In some cultures, there may be greater stigma associated with mental health concerns, making it more difficult for individuals to seek help. Additionally, the economic and social factors that contribute to burnout may vary depending on the cultural context. Therefore, it is important to understand the specific cultural factors that contribute to burnout in a given context to effectively address the problem.

Recognizing employees suffering from occupational burnout is not implemented routinely, which impedes timely control and prevention in the workplace. Measuring occupational burnout using a traditional scale is susceptible to bias due to the fear of losing one’s job if recognized as burnout. Therefore, understanding the characteristics of occupational burnout as a proxy to identify staff at risk offers an opportunity for early prevention.

Existing studies analyzing characteristics of occupational burnout mainly used traditional statistical methods such as descriptive data analysis, regression models, analysis of variance (ANOVA), statistical testing, structural equation models, and so on (4–10). The limitation of such traditional statistical methodology is the incapability of capturing the relationship of high-dimensional features interacting with each other, which contributes to the complexity of occupational burnout. Besides, as staff working in the same corporation is interconnected, the above-mentioned conventional methods may ignore the social network characteristics shared among them with or without burnout.

To fill the aforementioned gaps, we introduced the network science approach and examined its potential in solving the sparse problems in high dimensional data. We assumed that employees at the same place of employment as the social network existed. Therefore, the proposed network science analysis methodology is valid and can draw social network features among all the staff. This study aims to test the network science approach in capturing features on occupational burnout from high dimensional data from a big corporation staff survey. We also aim to provide new insights for occupational burnout prevention gained from the network science perspectives.

## 2. Materials and methods

### 2.1. Data source and study population characteristics

This study utilized a cross-sectional survey of corporate employees in China as its data source. A survey questionnaire was distributed online to all units and employees of the Guang Zhou Baiyun International Airport Company on November 7, 2021 and was collected on November 11, 2021, with a total of 6,689 questionnaires returned. After excluding the invalid samples, there were 5,794 (86.6%) completed surveys included in this analysis. The respondents came from different types of work attribute, including administrative (394/5,794, 6.8%), technicians (2,810/5,794, 48.5%), security officers (2,458/5,794, 42.4%), caterers, and information technology staff. Through a self-administered questionnaire, all respondents submitted information on five aspects, including demographic, work-related, health status, lifestyle, and occupational burnout. Occupational burnout was measured using Chinese version of the Maslach Burnout Inventory–General Survey (MBI-GS), which was one of the most widely used instruments to measure burnout. The MBI-GS is a 16-item, 7-point Likert-type scale self-report measure that evaluates attitudes regarding one’s work. It contains three subscales: emotional exhaustion, cynicism, and professional efficacy. Individual’s mean subscale scores were calculated and compared with the critical boundaries for the burnout profiles of the study group. Respondents with two or more subscale scores that were higher than the critical boundaries were considered as burnout, otherwise as not burnout.

### 2.2. Statistical testing methods

We first identified the relationship between occupational burnout and a number of characteristics that fall into four broad categories: demographic, work-related, health condition, and lifestyle.

For categorical variables, we used Chi-squared tests to compare differences between occupational burnout and non-occupational burnout participants. For continuous variables, due to non-normality, Wilcoxon rank sum tests were incorporated in our analysis. Social network inference.

From network science perspective, we assumed the staff who were working together at the same company shared a social network (11). We inferred the social network of all participants by proposing a Survey2Vector method. Our inference was based on homogeneous network, where nodes sharing similar attributes are more likely to link each other (12). Such similar attributes, in this study, refer to demographic, work-related, health status, and lifestyle (13–20).

We proposed Survey2Vector method first represents all variables of demographic, work-related, health status, and lifestyle, after standardizing, as a vector for each participant, defined as $X_{i}$ where i=1,2,…,5794. The dimension of each vector is n, which is the number of all variables of demographic, work-related, health status, and lifestyle. Each element lies in [0, 1] after standardizing. That is:


$$X_{i}=\left(x_{i1},x_{i2},\ldots ,x_{\mathrm{in}}\right)$$

$$x_{il}\in \left[0,1\right]\;for\;all\;i=1,2,\ldots ,5794\;and\;l=1,2,\ldots ,n.$$

The participants/employees were treated as nodes in a social network, then such each vector $X_{i}$ can indicate attributes of each node i. We further calculated the similarity $S_{ij}\in \left[0,1\right]$ between the node i and the node j by using the cosine similarity definition:


$$S_{ij}=\mathrm{similarity}=\cos\theta =\frac{X_{i}\cdot X_{j}}{\left||X_{i}||||X_{j}|\right|}=\frac{\sum_{l=1}^{n}x_{il}x_{jl}}{\sqrt{\sum_{l=1}^{n}\left(x_{il}{}^{2}\right)}\sqrt{\sum_{l=1}^{n}\left(x_{jl}{}^{2}\right)}}.$$

Therefore, we can obtain a cosine similarity matrix among all respondents (i.e., nodes) considering variables of demographic, work-related, health status, and lifestyle. This matrix M equals to an adjacency similarity matrix:


$$M=\left[S_{ij}\right].$$

Then, we set a threshold value t for determining the adjacency matrix $M^{\prime }$:


$$M^{\prime }=\left[S_{ij}^{\prime }\right].$$

$$S_{ij}^{\prime }=1,if\;S_{ij}\geq t.$$

$$S_{ij}^{\prime }=0,if\;S_{ij}<t.$$

This threshold value *t* was set according to the estimated average degree of each node (respondent) which was estimated by the human resource director of the company. Finally, we obtained the inferred social network based on the adjacency matrix $M^{\prime }.$

### 2.3. Exponential random graph model

In the above-inferred social network among all employees, an exponential random graph model (ERGM) is utilized to analyze how occupational burnout may affect the edge in this social network. ERGM has been widely used in social network analysis (21, 22). The assumption of ERGM is that the probability of each graph is proportional to some network measures in an exponential way. Specifically, the probability of observing the current network (*y*) can be parameterized in the following form (23, 24):


$$P\left(Y=y|X\right)=\frac{\exp\left\{\sum_{a=1}^{A}\theta_{a}^{T}g_{a}\left(y,X\right)\right\}}{\kappa }$$

where *X* is a matrix of attributes (covariate vector) for nodes and edges. *A* is the set of all network configurations. $g_{a}\left(y,X\right)$ refers to the network statistics corresponding to configuration *a*, and is determined by:


$$g_{a}\left(y,\;X\right)=\left\{\begin{array}{l} 1,\mathrm{the}\;\mathrm{configuration is observed in the network}\;y\\ 0,\;\;\mathrm{otherwise} \end{array}\right.$$

$\theta_{a}$ is the coefficient corresponding to configurations $g_{a}\left(y,X\right)$. κ is a normalizing term to satisfy the probability sum to 1.

Exponential random graph model estimates the prevalence of all configurations, thus can help us to explore the relationship between the occupational burnout status of nodes (employees) and the social network linkages. We adopt Markov Chain Monte Carlo Maximum Likelihood Estimation (MCMCMLE) to maximize the possibility of the observed network structure (25). To investigate the occupational burnout of each node, we further adopted two models: zero model and node-attribute model. The zero model, considering only the number of edges, was treated as a control group. The node-attribute model accounted for node attributes (i.e., occupational burnout of each node) on the base of the zero model. We presented the results of the model with lower AIC and BIC values.

## 3. Results

### 3.1. Statistical testing results

For demographic variables, age (*p* < 0.01) and education background (*p* < 0.01) were significantly associated with occupational burnout. For work-related variables, type of position (*p* < 0.01) was a significant factor for occupational burnout. High occupational burnout risk was most prevalent among aviation technician workers (37.8%). For health status variables, self-rated health status, hospital admission history in the last 3 years, arthritis, cardiovascular diseases, high blood lipid, breast diseases, and other chronic diseases were all associated with occupational burnout significantly (*p* < 0.01). For lifestyle variables, breakfast frequency, dairy consumption, salt-limiting tool usage, oil-limiting tool usage, vegetable consumption, pedometer (step counter) usage, consuming various types of food (in the past year), fresh fruit and vegetable consumption (in the past year), physical exercise participation (in the past year), limit salt consumption, limit oil consumption and keep weight were also significant correlated with occupational burnout (*p* < 0.01). More details of results can be found in the following Table 1.

**Table 1:** Characteristics of participants categorized by burnout risk.

Category	Variable	Overall (*N* = 5,794)	Burnout risk		Value of *p*
			Low (*n* = 5,066)	High (*n* = 728)	
Demographic	Age (years)^*^	33.0 (25–41)	33.0 (25–41)	31.0 (25–39)	< 0.01
	Female	2,175 (62.5)	1882 (37.1)	293 (40.2)	0.11
	Marital status				0.01
	Married	3,371 (58.2)	2,987 (59.0)	384 (52.7)	
	Unmarried	2,263 (39.1)	1946 (38.4)	317 (43.5)	
	Widowed	18 (0.3)	15 (0.3)	3 (0.4)	
	Divorced	142 (2.5)	118 (2.3)	24 (3.3)	
	Education background				< 0.01
	High school	1,253 (21.6)	1,127 (22.2)	126 (17.3)	
	College	2,728 (47.1)	2,359 (46.6)	369 (50.7)	
	University	1,661 (28.7)	1,440 (28.4)	221 (30.4)	
	Graduate	152 (2.6)	140 (2.8)	12 (1.6)	
Work related	Type of position				< 0.01
	Management	394 (6.8)	352 (6.9)	42 (5.8)	
	Profession	1,192 (20.6)	1,039 (20.5)	153 (21.0)	
	Aviation Technology	1863 (32.2)	1,588 (31.3)	275 (37.8)	
	General Technology	947 (16.3)	842 (16.6)	105 (14.4)	
	Assistant	1,398 (24.1)	1,245 (24.6)	153 (21.0)	
	Professional grade				0.08
	6–8	1,159 (26.2)	1,353 (26.7)	166 (22.8)	
	9–10	2,166 (37.4)	1858 (36.7)	308 (42.3)	
	11–13	1,234 (21.3)	1,084 (21.4)	150 (20.6)	
	14–15	370 (6.4)	328 (6.5)	42 (5.8)	
	16–18	341 (5.9)	300 (5.9)	41 (5.6)	
	19 or above	144 (2.5)	124 (2.4)	20 (2.7)	
	others	20 (0.4)	19 (0.4)	1 (0.1)	
Health and chronic diseases	Self-rated health status				< 0.01
	Healthy	1,202 (20.8)	1,093 (21.6)	109 (15.0)	
	Relatively healthy	1,208 (39.8)	2,107 (41.6)	201 (27.6)	
	Sub-health	1930 (33.3)	1,605 (31.7)	325 (44.6)	
	Unhealthy	354 (6.1)	261 (5.2)	93 (12.8)	
	Hospital admission in the last 3 years				< 0.01
	Two or more times	182 (3.1)	144 (2.8)	38 (5.2)	
	Once	654 (11.3)	577 (11.4)	77 (10.6)	
	Never	4,958 (85.6)	4,345 (85.8)	613 (84.2)	
	Arthritis				< 0.01
	Yes	1,125 (19.4)	936 (18.5)	189 (26.0)	
	No	4,669 (80.6)	4,130 (81.5)	539 (74.0)	
	High blood pressure				0.09
	Yes	458 (7.9)	389 (7.7)	69 (9.5)	
	No	5,336 (92.1)	4,677 (92.3)	659 (90.5)	
	Cardiovascular diseases				< 0.01
	Yes	91 (1.6)	66 (1.3)	25 (3.4)	
	No	5,703 (98.4)	5,000 (98.7)	703 (96.6)	
	High blood lipid				< 0.01
	Yes	506 (8.7)	412 (8.1)	94 (12.9)	
	No	5,288 (91.3)	4,654 (91.9)	634 (87.1)	
	Breast diseases				< 0.01
	Yes	283 (4.9)	226 (4.5)	57 (7.8)	
	No	5,511 (95.1)	4,840 (95.5)	671 (92.2)	
	Overweight				0.07
	Yes	1,001 (17.3)	858 (16.9)	143 (19.6)	
	No	4,793 (82.7)	4,208 (83.1)	585 (80.4)	
	Other chronic diseases				< 0.01
	Yes	804 (13.9)	651 (12.9)	153 (21.0)	
	No	4,990 (86.1)	4,415 (87.1)	575 (79.0)	
Lifestyle	Breakfast frequency^*^				< 0.01	
	Almost everyday	3,190 (55.1)	2,868 (56.6)	322 (44.2)		
	3 days or more per week	2,123 (36.6)	1821 (35.9)	302 (41.5)		
	Less than 1 day per week	481 (8.3)	377 (7.4)	104 (14.3)		
	Smoked food consumption				0.04	
	Often	431 (7.4)	360 (7.1)	71 (9.8)		
	Sometimes	3,925 (67.7)	3,443 (68.0)	482 (66.2)		
	Almost never	1,438 (24.8)	1,263 (24.9)	175 (24.0)		
	Diary consumption				< 0.01	
	< 200 ML/day	2,590 (44.7)	2,261 (44.6)	329 (45.2)		
	200–300 ML/day	1,072 (18.5)	976 (19.3)	96 (13.2)		
	> 400 ML/day	178 (3.1)	158 (3.1)	20 (2.7)		
	Almost never	1954 (33.7)	1,671 (33.0)	283 (38.9)		
	Salt-limiting tool usage				< 0.01	
	Almost never	3,853 (66.5)	3,329 (65.7)	524 (72.0)		
	Sometimes	985 (17.0)	885 (17.5)	100 (13.7)		
	Often	411 (7.1)	370 (7.3)	41 (5.6)		
	Always	545 (9.4)	482 (9.5)	63 (8.7)		
	Change of taste				0.04	
	Salty	638 (11.0)	539 (10.6)	99 (13.6)		
	Light	1,461 (25.2)	1,273 (25.1)	188 (25.8)		
	No change	3,695 (63.8)	3,254 (64.2)	441 (60.6)		
	Oil-limiting tool usage				< 0.01	
	Almost never	4,209 (72.6)	3,637 (71.8)	572 (78.6)		
	Sometimes	803 (13.9)	726 (14.3)	77 (10.6)		
	Often	368 (6.4)	332 (6.6)	36 (4.9)		
	Always	414 (7.1)	371 (7.3)	43 (5.9)		
	Change of oil consumption				0.03	
	More	469 (8.1)	395 (7.8)	74 (10.2)		
	Less	1,338 (23.1)	1,189 (23.5)	149 (20.5)		
	No change	3,987 (68.8)	3,482 (68.7)	505 (69.4)		
	Vegetable consumption				< 0.01	
	Almost never	442 (7.6)	379 (7.5)	63 (8.7)		
	1–3 times/week	1,266 (21.9)	1,094 (21.6)	172 (23.6)		
	4–6 times/week	1,088 (18.8)	977 (19.3)	111 (15.2)		
	1 time/day	961 (16.6)	858 (16.9)	103 (14.1)		
	2 times/day	876 (15.1)	779 (15.4)	97 (13.3)		
	3 times/day	150 (2.6)	132 (2.6)	18 (2.5)		
	Do not remember	1,011 (17.4)	847 (16.7)	164 (22.5)		
	Pedometer (step counter) usage				< 0.01	
	Often	1,615 (27.9)	1,435 (28.3)	180 (24.7)		
	Sometimes	1,492 (25.8)	1,333 (26.3)	159 (21.8)		
	Never	2,687 (46.4)	2,298 (45.4)	389 (53.4)		
	Weight status				0.03	
	Underweight	778 (13.4)	663 (13.1)	115 (15.8)		
	Normal	2,192 (37.8)	1939 (38.3)	253 (34.8)		
	Overweight	2,348 (40.5)	2061 (40.7)	287 (39.4)		
	Do not know	476 (8.2)	403 (8.0)	73 (10.0)		
	Smoking (in the past year)				0.08	
	Everyday	1,128 (19.5)	973 (19.2)	155 (21.3)		
	Sometimes	760 (13.1)	682 (13.5)	78 (10.7)		
	Never	3,906 (67.4)	3,411 (67.3)	495 (68.0)		
	Consumed various types of food (in the past year)				< 0.01	
	Yes	4,095 (70.7)	3,629 (71.6)	466 (64.0)		
	No	1,699 (29.3)	1,437 (28.4)	262 (36.0)		
	Fresh fruit and vegetable consumption (in the past year)				< 0.01	
	Enough	3,027 (52.2)	2,701 (53.3)	326 (44.8)		
	Not enough	2,767 (47.8)	2,365 (46.7)	402 (55.2)		
	Oily food consumption (in the past year)				0.02	
	Often	1,372 (23.7)	1,174 (23.2)	198 (27.2)		
	Not often	4,422 (76.3)	3,892 (76.8)	530 (72.8)		
	Physical exercise participation (in the past year)				< 0.01	
	Often	2,657 (45.9)	2,400 (47.4)	257 (35.3)		
	Not often	3,137 (54.1)	2,666 (52.6)	471 (64.7)		
	Limit salt consumption				< 0.01	
	Yes	3,633 (62.7)	3,239 (63.9)	394 (54.1)		
	No	2,161 (37.3)	1827 (36.1)	334 (45.9)		
	Limit oil consumption				< 0.01	
	Yes	2,855 (49.3)	2,543 (50.2)	312 (42.9)		
	No	2,939 (50.7)	2,523 (49.8)	416 (57.1)		
	Keep weight				< 0.01	
	Yes	3,009 (49.3)	2,702 (53.3)	307 (42.2)		
	No	2,785 (50.7)	2,364 (46.7)	421 (57.8)		
	Walk time (min/week)	80.0 (60–180)	76.5 (60–180)	80 (60–264)	0.11

* median (interquartile range, IQR) were reported for numerical variables; count (%) were reported for categorical variables.

### 3.2. The inferred social network

**Figure 1:**
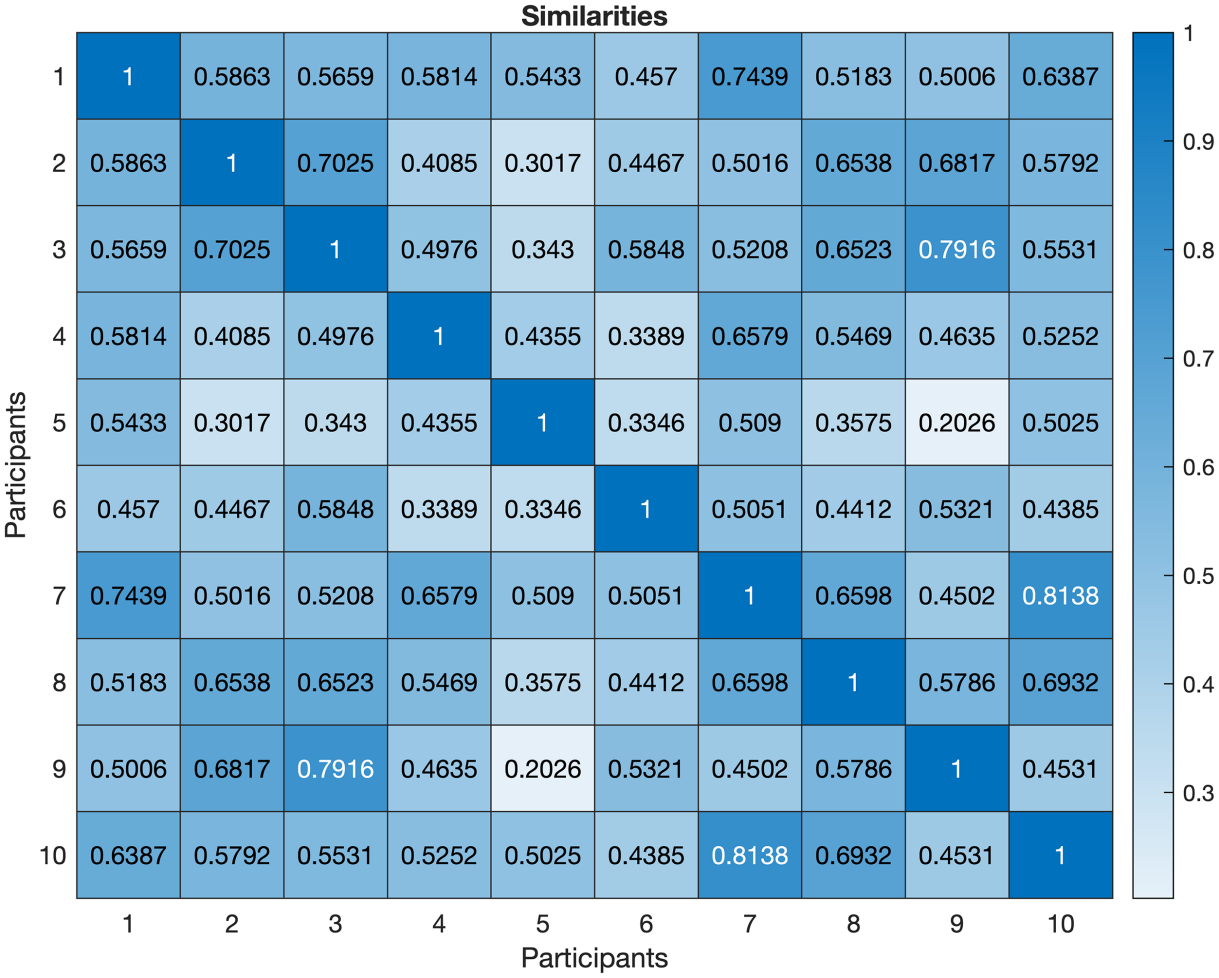
Similarity matrix for the first ten participants.

Following the steps outlined in the preceding method section, we obtained the adjacency similarity matrix M, which is illustrated in Figure 1 for the first 10 participants (employees).

**Figure 2:**
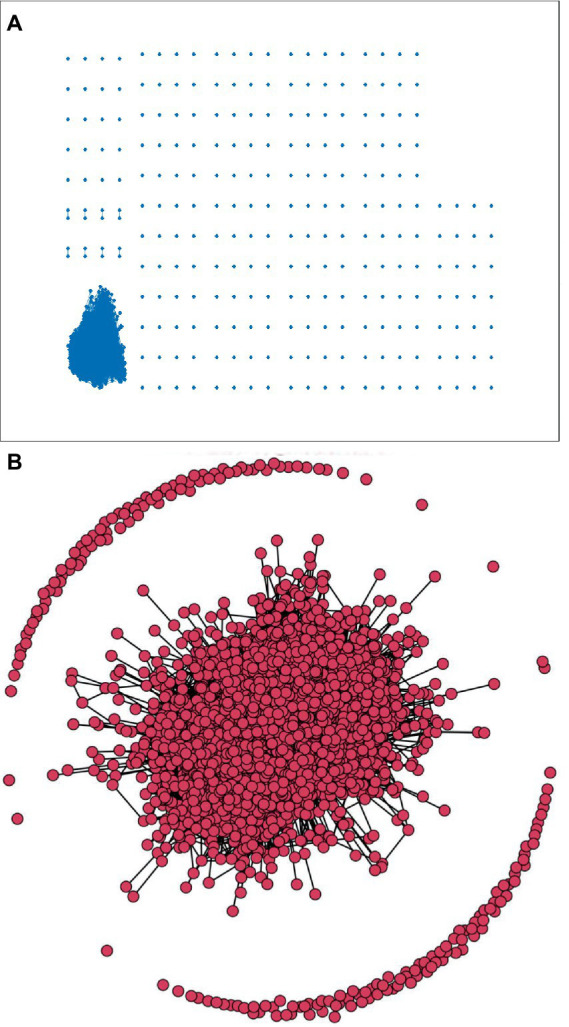
The inferred colleagues’ social network for all participants **(A)** Sparse and **(B)** Densely.

By setting the threshold value t=0.7799, we obtained our inferred social network, which is shown in the following Figure 2. The Figure 2A presents the inferred colleagues’ social network in a sparse way, while the Figure 2B presents the inferred colleagues’ social network densely.

### 3.3. Exponential random graph model results

The following Table 2 displays the estimated values of coefficients and statistical test results for ERGM model on the association between the occupational burnout and the inferred social network.

**Table 2:** Estimated values of coefficients and statistical test results for exponential random graph model (ERGM) model.

Configurations	Estimated value (Std. Error)	*Z*-value	*p*-Value	AIC	BIC
edges	−4.65 (0.01)	−371.59	<1*e*–04^***^		
burnout	−0.14 (0.01)	−12.53	<1*e*–04^***^		
nodematch.burnout	0.09 (0.01)	6.94	<1*e*–04^***^	1,851,837	1,851,881

Denote: ^***^p < 0.001; ^**^p < 0.01; ^*^p < 0.05.

The results of the ERGM can be interpreted as follows. If two nodes (employees) were both in high risk of occupational burnout, they were more likely to be connected by an edge in our inferred social network. Similarly, if these two nodes (employees) both had a low risk of occupational burnout, they were also more likely to be linked through an edge in this inferred social network. In other words, whether or not two employees shared the same occupational burnout status significantly mattered in this social network (*p* < 0.001).

## 4. Discussion

This study is the first to employ a network science approach to explore the social, lifestyle, and health status characteristics as a proxy to identify personnel at risk of occupational burnout. Our findings suggest that occupational burnout populations can be grouped based on distinct social, behavioral, and biological characteristics. Those with the same occupational burnout status (e.g., low or high) are more likely to be connected *via* an edge in an inferred social network. This study extends our knowledge in identifying occupational burnout from a network science perspective. Instead of employing a stigmatized and notorious scale, we can deduce a person’s burnout status based on the social network to which he or she belongs. Meanwhile, our study also shreds lights on the promoting workplace health in a network scale.

In this study, the association between occupational burnout and demographic, work-related, health & chronic disease, and lifestyle characteristics was identified. These associated factors include age, educational background, type of position, hospitalization history, cardiovascular illnesses, consumption of fresh fruits and vegetables, and physical activity involvement, among others. Our findings are consistent with previous studies considering similar factors across several industries (26–31). Consequently, the inferred social network constructed using these features makes good sense, and the inferences made within the network are reliable. In addition to corroborating current research, our findings demonstrate that mental health issues and psychological syndromes can spread across a social network (32–36). Our findings suggest that occupational burnout may also be infectious through the colleague’s social network.

Potential implications usually lie in interventions for occupational burnout based on the identified factors (37). For example, the company can provide more fresh fruits and vegetables in the staff canteen and may provide fitness areas for employee use (38, 39). Based on the findings of this study from the perspective of network science, the company may encourage employees whose occupational burnout status is low to make more friends or contacts, or work together with colleagues on some projects. For employees with a high level of occupational burnout, the organization may provide support by offering confidential assessments, short-term counselling, etc., and assign them more independent tasks.

The major limitation of this work is that the social network for occupational burnout ERGM analysis was inferred based on associated factors, such as demographics, work-related conditions, health and chronic diseases, and behaviors. Even though these factors have been proven to be associated with occupational burnout, the accuracy of the conclusions derived from this social network have not been verified. Due to heterogeneity of the type of business and nature of work, social network established in one workplace may not be transferable to others. In future work, we will try to use various indicators of the subjects (demographic, work-related, health status, lifestyle) to predict occupational burnout and further explore its practical value.

In conclusion, this study demonstrated the feasibility of identifying occupational burnout through an inferred social network and shed new insights on the prevention of occupational burnout from a network science perspective. The outcomes of network inference should be validated in the real-world setting.

## Data availability statement

The raw data supporting the conclusions of this article will be made available by the authors, without undue reservation.

## Ethics statement

The studies involving human participants were reviewed and approved by Ethics Committee of Guangdong Second Provincial General Hospital. The patients/participants provided their written informed consent to participate in this study.

## Author contributions

JL, WMC, and WBC conceived this study. FJ, ZX, and WBC were involved in study design and methodology. MC, HR, and ZX participated in data cleaning and analysis. FJ made the original draft preparation. MC, JZ, and HZ critically revised this manuscript. JL, WMC, and WBC supervised and validated the study. All authors contributed to the article and approved the submitted version.

## Funding

This work was supported by the Key-Area Research and Development Program of Guangdong Province (2020B0101130020), and by the Guangzhou Science and Technology Project (No. 2023A03J0286 and No. 2023A04J1715).

## Conflict of interest

JL was employed by the company Guangzhou Baiyun International Airport Co. Ltd.

The remaining authors declare that the research was conducted in the absence of any commercial or financial relationships that could be construed as a potential conflict of interest.

## Correction note

A correction has been made to this article. Details can be found at: 10.3389/fpsyt.2025.1642874.

## Publisher’s note

All claims expressed in this article are solely those of the authors and do not necessarily represent those of their affiliated organizations, or those of the publisher, the editors and the reviewers. Any product that may be evaluated in this article, or claim that may be made by its manufacturer, is not guaranteed or endorsed by the publisher.
