# Correction: Social, lifestyle, and health status characteristics as a proxy for occupational burnout identification: a network approach analysis

**DOI:** 10.3389/fpsyt.2025.1642874

**Published:** 2025-06-23

**Authors:** Fengshi Jing, Mengyuan Cheng, Jing Li, Chaocheng He, Hao Ren, Jiandong Zhou, Hanchu Zhou, Zhongzhi Xu, Weiming Chen, Weibin Cheng

**Affiliations:** ^1^ Institute for Healthcare Artificial Intelligence Application, Guangdong Second Provincial General Hospital, Guangzhou, China; ^2^ UNC Project-China, UNC Global, School of Medicine, The University of North Carolina, Chapel Hill, NC, United States; ^3^ Guangzhou Baiyun International Airport Co., Ltd, Guangzhou, China; ^4^ School of Information Management, Wuhan University, Wuhan, China; ^5^ Nuffield Department of Medicine, University of Oxford, Oxford, United Kingdom; ^6^ School of Traffic and Transportation Engineering, Central South University, Changsha, China; ^7^ School of Data Science, City University of Hong Kong, Kowloon, Hong Kong SAR, China; ^8^ School of Public Health, Sun Yat-Sen University, Guangzhou, China; ^9^ Health Medicine Department, Guangdong Second Provincial General Hospital, Guangzhou, China

**Keywords:** occupational burnout, network science, health management, exponential random graph model, social networks

An incorrect **Funding** statement was provided. The authors previously used the funding proposal application numbers instead of grant numbers. The statement previously said:

“This work was supported by the Key-Area Research and Development Program of Guangdong Province (2020B0101130020), and by the Guangzhou Science and Technology Project (No. SL2022A03J00781 and No. SL2022A04J01130)”.

The corrected statement appears below:

“This work was supported by the Key-Area Research and Development Program of Guangdong Province (2020B0101130020), and by the Guangzhou Science and Technology Project (No. 2023A03J0286 and No. 2023A04J1715)”.

The original version of this article has been updated.

